# Noninvasive Prenatal Detection for Pathogenic CNVs: The Application in α-Thalassemia

**DOI:** 10.1371/journal.pone.0067464

**Published:** 2013-06-28

**Authors:** Huijuan Ge, Xuan Huang, Xuchao Li, Shengpei Chen, Jing Zheng, Haojun Jiang, Chunlei Zhang, Xiaoyu Pan, Jing Guo, Fang Chen, Ning Chen, Qun Fang, Hui Jiang, Wei Wang

**Affiliations:** 1 Fetal Medicine Center, Department of Obstetrics and Gynecology, First Affiliated Hospital of Sun Yat-sen University, Guangzhou, Guangdong Province, China; 2 BGI-Shenzhen, Shenzhen, China; 3 Shenzhen Clinical Laboratory, Shenzhen, China; 4 Department of Biomedical Engineering, Southeast University, Nanjing, China; 5 School of Bioscience and Bioengineering, South China University of Technology, Guangzhou, Guangdong Province, China; 6 Department of Biology, University of Copenhagen, Copenhagen, Denmark; Institute of Molecular Medicine, Taiwan

## Abstract

**Background:**

The discovery of cell free fetal DNA (cff-DNA) in maternal plasma has brought new insight for noninvasive prenatal diagnosis. Combining with the rapidly developed massively parallel sequencing technology, noninvasive prenatal detection of chromosome aneuploidy and single base variation has been successfully validated. However, few studies discussed the possibility of noninvasive pathogenic CNVs detection.

**Methodology/Principal Findings:**

A novel algorithm for noninvasive prenatal detection of fetal pathogenic CNVs was firstly tested in 5 pairs of parents with heterozygote α-thalassemia of Southeast Asian (SEA) deletion using target region capture sequencing for maternal plasma. Capture probes were designed for α-globin (HBA) and β-globin (HBB) gene, as well as 4,525 SNPs selected from 22 automatic chromosomes. Mixed adaptors with 384 different barcodes were employed to construct maternal plasma DNA library for massively parallel sequencing. The signal of fetal CNVs was calculated using the relative copy ratio (RCR) of maternal plasma combined with the analysis of *R*-score and *L*-score by comparing with normal control. With mean of 101.93× maternal plasma sequencing depth for the target region, the RCR value combined with further R-score and L-score analysis showed a possible homozygous deletion in the HBA gene region for one fetus, heterozygous deletion for two fetus and normal for the other two fetus, which was consistent with that of invasive prenatal diagnosis.

**Conclusions/Significance:**

Our study showed the feasibility to detect pathogenic CNVs using target region capture sequencing, which might greatly extend the scope of noninvasive prenatal diagnosis.

## Introduction

Over thousands of monogenetic diseases are reported with known phenotype and molecular mechanism with various incidences in different regions. Most monogenic diseases are caused by single base variations, indel or copy number variations (CNVs). Thalassemia is one of most common monogenetic diseases in the south of China and Southeast Asia. The genetic survey showed the frequency of carrier achieves 26.9% forα-thalassemia and 19.9% for β-thalassemia in Guangxi Zhuang Autonomous Region [1]. Another study in 471 children with α-thalassemia found the Southeast Asian (SEA) types is a major type of α-thalassemia gene deletion with 75.3% of frequency [2]. To decrease the incidence of these monogenetic diseases, prenatal diagnosis is wildly used as the most efficient approach in clinical practices. But traditional sampling approaches for prenatal diagnosis such as amniocentesis and chorionic villus sampling (CVS) are invasive and therefore might increase the risk of miscarriage [3].

The discovery of cell free fetal DNA (cff-DNA) in maternal plasma opens the door for noninvasive prenatal diagnosis [4]. However, the detection of fetal DNA is very difficult for the vast background of maternal DNA [5]. Up to now, several studies have been reported by different groups and demonstrate the high accuracy of noninvasive prenatal detection of fetal chromosome aneuploidy [6], [7]. Moreover, whole genome sequencing (WGS) of maternal plasma DNA can recover the fetal specific allele with over 95% of accuracy, presenting the possibility of monogenetic detection by maternal plasma [8]. However, the extremely high cost of whole genome deep sequencing restricts its application in clinic practices. Target region deep sequencing, which could achieve deep sequencing for the interested region with lower cost, is an alternative approach to detect monogenetic disease noninvasively [9]. Lo et al. developed an algorithm for noninvasive prenatal diagnosis of fetal monogenetic diseases using target region capture sequencing for single base variations and indels [10]. Using this method, two fetuses were correctly diagnosed to be carriers of β-thalassemia. However, there is still lack of effective approaches to noninvasively detect pathogenic CNVs for fetus, which is one of the most common causes in monogenetic diseases.

In this study, we developed a novel method for pathogenic CNVs detection using target region sequencing for maternal plasma. With much lower cost compared with whole genome sequencing, a suspected SEA type of homozygous α-thalassemia deletion in a fetus was successfully detected using our method. It was the first time for noninvasive prenatal detection of fetal small size CNVs from maternal heterozygous background.

## Results

### Clinical Sample and Data Production

Five couples both detected to be SEA type of α-thalassemia deletion carriers was recruited with informed written consent. Theoretically the fetus has a possibility of 25% to be Bart’s Hydrops Syndrom (–SEA/−SEA), Cordocentesis or amniocentesis was performed for each pregnant woman to conduct clinical diagnosis using gap-PCR. Maternal plasma was isolated for noninvasive prenatal test before cordocentesis and amniocentesis. Additionally, 12 health participants and 6 pregnant women normal in α-thalassemia were enrolled as normal control for genomic DNA (g-DNA) and plasma DNA respectively with informed written consent.

We performed target region capture sequencing for a total of 15 positive g-DNA samples and 5 positive maternal plasma samples. The g-DNA data of parents were combined with maternal plasma data for fetal CNV detection, and the fetal g-DNA data from cord blood or amniotic fluid sample was taken as feasibility control of this method. We obtained a median of 26.45×10^6^ raw reads for g-DNA samples. After basic data processing, the effective sequencing depth for g-DNA varied from 104.15× to 226.32×, corresponding 93.23% to 98.67% of coverage for target region (Table 1).

**Table 1:** Performance of target region capture sequencing.

Sample	Reads (M)	Data (MB)	Alignment (%)	Coverage (%)	Depth
MO-1	3.05	0.27	91.39	93.57	135.92
FA-1	2.37	0.21	88.77	95.30	104.15
FT-1	2.71	0.24	89.05	93.23	124.62
MP-1	128.89	11.59	93.58	95.63	203.21
MP-1(Duo-index)	128.89	11.59	93.58	95.66	279.91
MO-2	30.35	2.73	92.10	98.36	174.87
FA-2	22.09	1.99	92.90	97.14	135.81
FT-2	33.92	3.05	89.36	98.51	176.90
MP-2	141.91	12.78	95.34	98.61	62.28
MO-3	30.83	2.77	93.14	98.36	131.22
FA-3	33.04	2.97	93.31	97.43	148.44
FT-3	22.66	2.04	92.99	98.32	104.64
MP-3	145.86	13.12	95.28	98.68	44.75
MO-4	39.81	3.58	91.82	98.67	226.32
FA-4	28.02	2.52	92.19	97.30	176.12
FT-4	28.75	2.59	89.97	97.34	117.40
MP-4	188.27	16.94	89.16	97.56	93.81
MO-5	34.61	3.46	94.65	98.61	174.67
FA-5	43.09	4.31	94.20	97.27	180.79
FT-5	41.39	4.14	91.54	97.46	176.58
MP-5	134.99	12.14	95.24	97.11	28.91

For plasma DNA samples, we obtained a median of 147.98×10^6^ raw reads. We applied a double-indexed method to recover part of false duplication reads besides the conventional site-recognition method in one plasma sample. 95.66% of coverage was obtained in double-indexed method, compared with 95.63% of conventional site-recognition method (Table 1). As expected, the depth of target region displayed a significant improvement using new approach from 203.21× to 279.91×, implying 37.74% of increasing of useful reads. For the rest maternal plasma DNA samples, we got an effective sequencing depth varied from 28.91× to 93.81×using traditional data processing mode, corresponding 97.11% to 98.68% of coverage in the target region (Table 1). For control samples, the data production was showed in Table S1.

### Cff-DNA Concentration Estimation

**Figure 1:**
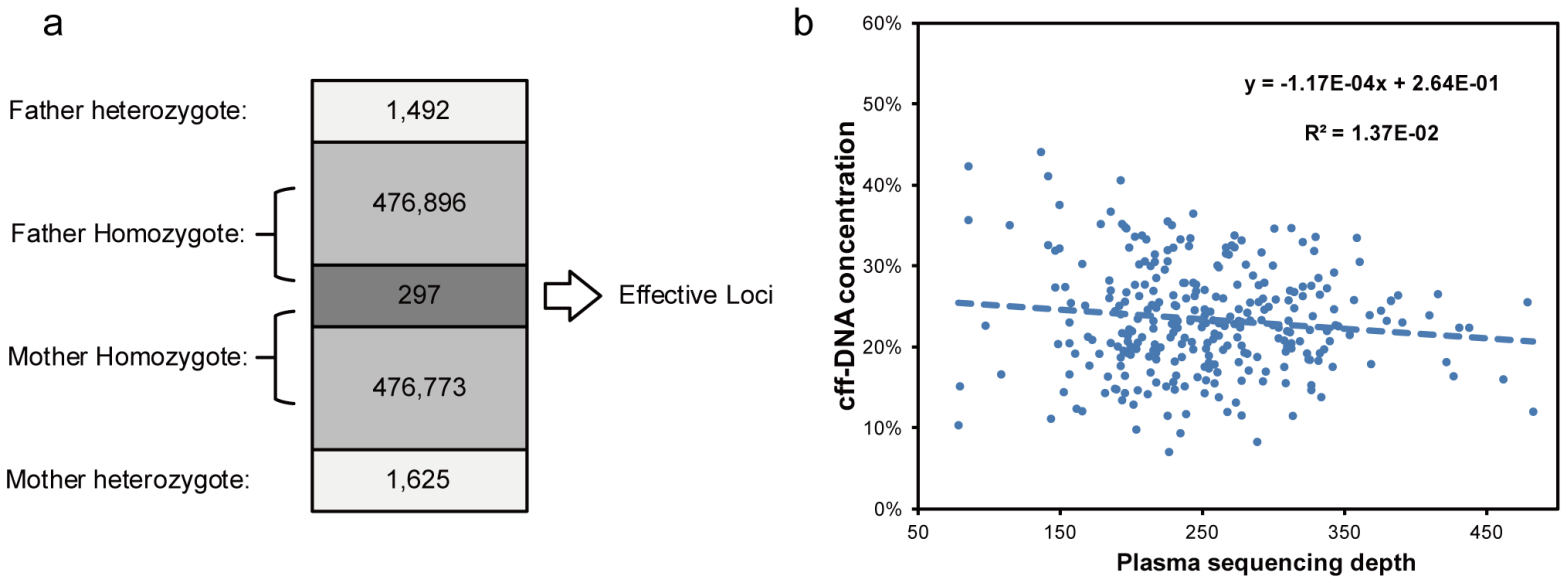
Fetal concentration calculation in maternal plasma. Fetal concentration in maternal plasma was calculated using both paternal and maternal homozygous but different type of loci. (a) Gene typing result of 478,685 loci were acquired for both parents. A total of 476,896+297 loci were homozygous for the father and 476,773+297 for the mother. There were 297 loci was homozygous in both parents but with different type and were used for fetal concentration calculation in maternal plasma. (b) Cff-DNA concentration and sequencing depth for each of the 297 loci.

The concentration of cff-DNA in maternal plasma was a key parameter in our method. Using the Bayesian model, we firstly called the overall loci of maternal and paternal gDNA, and select the homozygous locus for further analysis. For these five families, we finally isolated 54 to 706 eligible homozygous SNPs with different type of allele. Then, we estimated the cff-DNA concentration in each maternal plasma using these SNPs, which was 23.41%, 14.29%, 32.27%, 20.28%, and 7.90% (Figure 1 and Table 2). Our data showed a great fluctuation of cff-DNA concentration among these sites. The lowest value in family F-1 was only 7.09% while it was up to 63.53% in the highest site, with a median of 23.41% in the maternal plasma. Moreover, our data also showed that there was no relationship between fetal concentration fluctuation and the sequencing depth (Figure 1b).

**Table 2:** SNP calling and cff-DNA concentration.

Family	Father		Mother		Effective loci	Gestational age	cff-DNA Conc. (%)
	heterozygote	homozygote	heterozygote	homozygote			
F-1	1,492	477,193	1,615	477,070	297	30^+6^	23.41±6.95
F-2	2,760	1,009,442	2,970	1,009,232	553	19^+1^	14.29±7.76
F-3	1,613	579,525	1,777	579,361	282	20^+3^	32.27±13.95
F-4	2,843	1,167,315	3,262	1,166,896	706	17^+2^	20.28±8.01
F-5	190	73,498	223	73,465	54	19	7.90±7.23

The color-coded points and lines showed the RCRs distribution of the target regions on HBA gene. RCR value of 0 means a homozygous deletion, 0.5 mean a heterozygous deletion and 1.0 mean normal. The orange broken line represented the mean RCR in maternal plasma. (Gray, 4 plasma control samples respectively; Blue, g-DNA from paternal blood; Red, g-DNA from maternal blood cells; Orange, cell free DNA from maternal plasma; Green, g-DNA from fetal amniotic fluid). (a, F-2; b, F-3; c, F-4; d, F-5).

### Pathogenic CNVs Detection

**Figure 2:**
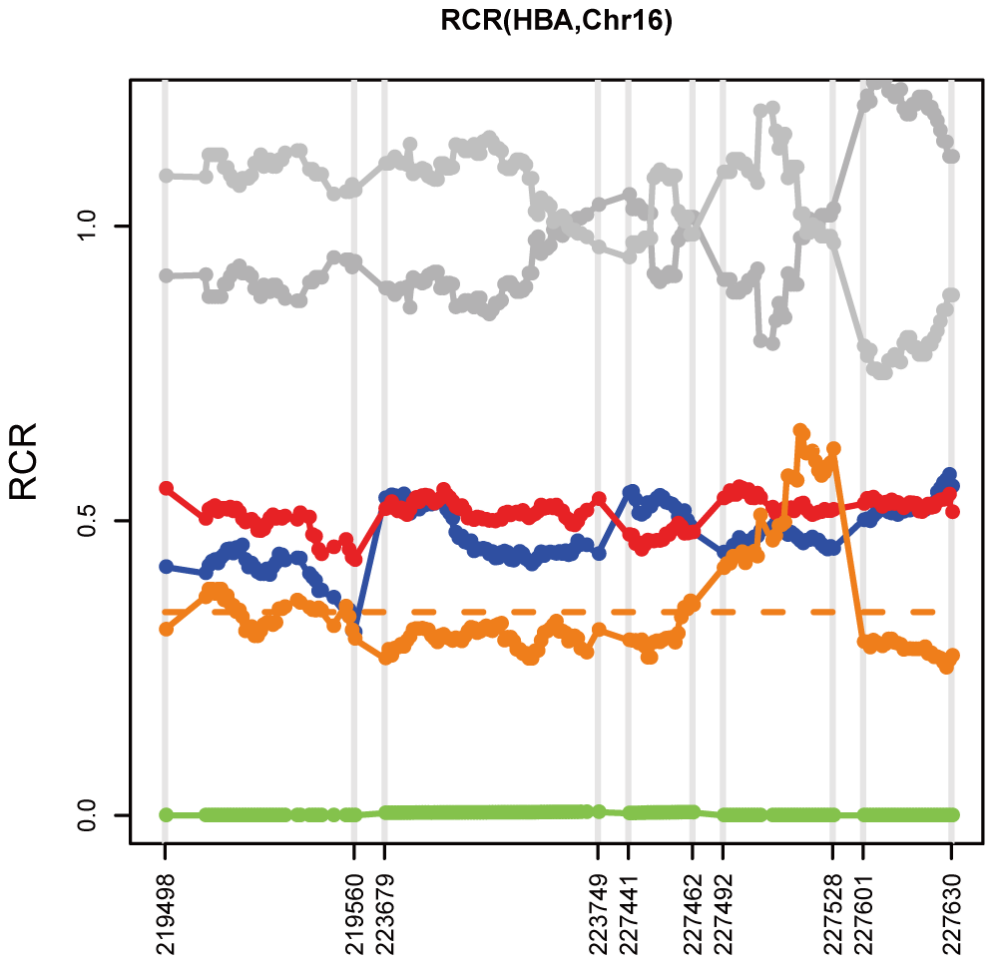
The RCR distribution of target region on HBA. The color-coded points and lines showed the RCRs distribution of the target regions on HBA gene in F-1. RCR value of 0 mean a homozygous, 0.5 mean a heterozygous and 1.0 mean normal. The orange broken line represented the mean RCR in maternal plasma. (Gray, 2 control samples respectively; Blue, g-DNA from paternal blood; Red, g-DNA from maternal blood cells; Orange, cell free DNA from maternal plasma; Green, g-DNA from fetal amniotic fluid).

The relative copy ratio (RCR) value, which revealing the relative depth of a sample, was calculated in each target region and directly used as indicator for CNV detection. The average value of RCR (ARCR) on chromosome 16 from 215,400 to 234,700 for paternal and maternal g-DNA was varied from about 0.43 to 0.58, which indicated a heterozygous SEA type of α-thalassemia deletion (Figure 2). In addition, the ARCR value for fetal g-DNA that close to 0 would strongly suggest a homozygous deletion in the same region, close to 0.5 suggest a heterozygous deletion, and close to 1 suggest no deletion. This result was consistent with the clinical result of gap-PCR. The RCR values of other four families were showed in Figure S1.

To further ensure the genotypes of each region, we defined two statistics, *R*-score and *L*-score, to detect the pathogenic CNVs. *R*-score was defined as the negative logarithmic of the quotient between the rank of test region and the total number of target regions. It revealed the deviation of certain region in the tested sample. In the case of deletion, we set the cutoff as 1.3 (α = 0.05), the larger *R*-score stands for a higher significance. Meanwhile, we also recruited a binary assumption strategy to determine the CNV genotypes. The *L*-score was the natural logarithm of odd ratio between these two statistics tests of mutually exclusive assumptions, representing the significance of expected CNV genotype by comparing to the control samples (Material and Methods). In the deletion region mentioned above, both the R-score and L-score were calculated.

**Figure 3:**
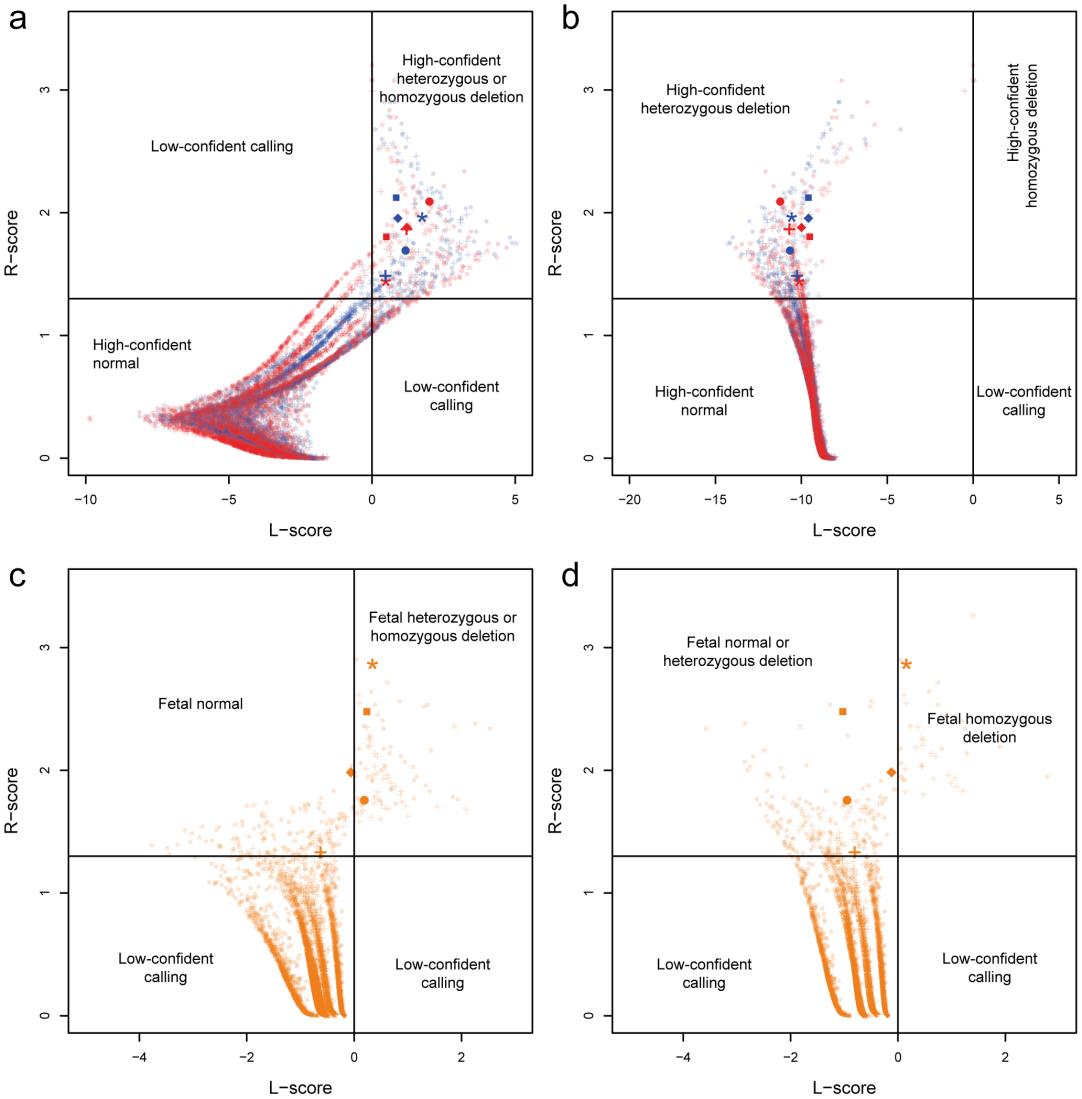
The R-sore and L-score of parental CNV detection using genome DNA and noninvasive fetal CNV detection using maternal plasma. (a, b), The R-score (y-axis) and L-score (x-axis) of each target region using paternal (blue) and maternal (red) g-DNA were illustrated as scatter points. The quadrants of normal (copy number = 2), heterozygous deletion (copy number = 1), homozygous deletion (copy number = 1) and low-confident calling are labeled in the figure. The L-score in (a) was the odd ratio of normal and heterozygous deletion, and in (b) was the odd ratio of heterozygous deletion and homozygous deletion. (c, d), The R-score (y-axis) and L-score (x-axis) of each target region using maternal plasma (orange) were illustrated as scatter points. The quadrants of fetal normal (copy number = 2), fetal heterozygous deletion (copy number = 1), fetal homozygous deletion (copy number = 0) and low-confident calling are labeled in the figure. The L-score in (c) was the odd ratio of fetal normal and fetal heterozygous deletion, and in (d) was the odd ratio of fetal heterozygous deletion and fetal homozygous deletion. The shapes of scatters represented the different family. (“*”, F-1; “+” F-2; Square, F-3; Round, F-4; Diamond, F-5.).

We firstly detected that all of the five pair of parents located in the quadrant of heterozygous deletion, one of the fetuses located in the quadrant of homozygous deletion, two located in the heterozygous quadrant, and two located in the normal quadrant (Figure 3a, b), which was consistent with the result from RCR calculation.

Combined with the information of fetal concentration, we also classified all five samples into three subgroups, one located in the quadrant of homozygous deletion, two located in the heterozygous quadrant, and two located in the normal quadrant (Figure 3c, d), which was consistent with the result of fetal gDNA. Our data showed that the fetal pathogenic deletion could be detected using our bioinformatics method through maternal plasma target region sequencing.

## Discussion

Since the discovery of cff-DNA in maternal plasma, it has long been expected to noninvasive prenatal detection for fetal aneuploidies, even single base mutations and pathogenic CNVs. Until recently the noninvasive prenatal detection for chromosome aneuploidy comes to reality with the rapid development of massively parallel sequencing [6], [7] which also makes pathogenic CNVs detection possible. In this study, we developed a novel method based on target region capture sequencing to detect the fetal pathogenic CNVs noninvasively.

In our study, the target region was less than 1.5M bp, about 1/2,000 of human genome. With a mean depth of lower than 300× in the target region of maternal plasma, the fetal deletion status in HBA region was successfully detected using our method. Since it would cost about $41 for generating 1GB sequencing reads at present [11], our method was very cost effective. Of course, this cost would be further reduced as the innovation of the Third-generation sequencing technology.

The limited starting DNA fragment in plasma is a key obstacle to get enough deep sequencing data for distinguishing fetal genotype. Here we set up a double-indexed DNA library construction approach and determined the duplicated sequencing reads by the barcodes at each ends of the reads, which could dramatically increase the ratio of useful sequencing reads. This approach has been reported to effectively reduce bias produced during amplification [12]. Double indexing has been proved to be extremely effective to overcome inaccuracies in multiplex sequencing on the Illumina sequencer platform [13]. Conventional site-recognition method filters duplication reads with same start point and end point introduced by over amplification in PCR procedure, which could mistakenly lose part of originally same reads or keep the real duplicated reads if there was a sequencing error at the end of the reads. The double-indexed approach showed a 37.74% of increasing for effective depth of target region, displaying its great potential to overcome the limitation of starting materials in plasma.

Fetal concentration in maternal plasma was another important factor to make it difficult for fetal variation detection. As our data showed that the fetal DNA was only a tiny part in maternal plasma and the concentration fluctuated greatly in different sites. As a result, the fetal CNVs with small size would be much more difficult to be detected, especially for maternal sauce variations. To solve this problem, we developed a new bioinformatics algorithm that based on plasma high-depth sequencing. In this study, we employed target region capture sequencing instead of WGS as reported by previous studies [9]. The advantage of target region capture sequencing is cost-effective and labor-saving. In our study only one tenth of sequencing data was required to generate for analysis compared with WGS, but the sequencing depth for the target region was ten times higher. Most importantly, it is feasible to get the exactly bound and size of CNVs.

It is very difficult to directly detect small size of fetal CNVs from maternal plasma, especially in maternal heterozygous background. Our data has showed great variation of the fetal concentration in maternal plasma and the variation won’t be eliminated with higher sequencing depth (Figure 1b). This variation will also greatly affect the accuracy of fetal CNVs detection. To solve this problem, we used 12 control samples to adjust the data variation in gDNA samples and 6 control samples to adjust the data variation in maternal plasma samples. Then we use the ARCR value to detect if there is a CNV and the *L*-score and *R*-score to further confirm the CNV status. At last, the size of the CNVs detected from the maternal plasma was decided by combing the result of the parents.

However, there were still limitations for our study. The maternal plasma samples were mostly from late pregnancy due to the inconvenient of sample collection, which showed a high concentration of fetal DNA. Although the fetal α-thalassemia deletion (–SEA/−SEA) status had been successfully detected, the method needs to be tested with more samples at earlier gestational age. To improve the detection accuracy, more control samples are needed in future study. Moreover, the capture specificity for plasma DNA was much lower than g-DNA, which might be related with its fragment size. Further experimental optimization is required in the future.

In conclusion, our study could figure out the fetal deletion related with α-thalassemia from the maternal heterozygous background. This result showed the feasibility of fetal pathogenic CNVs detection using target region capture sequencing of maternal plasma with our novel bioinformatics method.

## Materials and Methods

### Clinical Samples

Five couples were recruited from the First Affiliated Hospital of Sun Yatsen University with informed written consent. Both the pregnant women and their husbands were detected to be –SEA type of α-thalassemia deletion carriers by gap-PCR. To make a prenatal diagnosis, amniocentesis or cordocentesis was performed at 30^+6^, 19^+1^, 20^+3^, 17^+2^, and 19 gestational weeks respectively, and its result was considered as the golden standard traditionally for prenatal diagnosis. Twelve health participants and 6 pregnant women normal in α-thalassemia were enrolled as normal control with informed written consent. Also, this study was approved by the institutional review board of BGI-Shenzhen and conducted in accordance with the Declaration of Helsinki.

### Probes Design

The fetal pathogenic CNV detection was based on our novel bioinformatics method using maternal plasma target region sequencing. Considering the tremendous impact of cff-DNA concentration on fetal pathogenic CNVs detection, target region was consisted by two parts. The gene regions of α-globin (HBA) and β-globin (HBB) gene and 100 bp upstream and downstream of the gene region was firstly included, and then a total of 4,525 SNPs were selected to calculate the exact concentration of cff-DNA in maternal plasma. These SNPs were selected from 22 autosomes with the minor allele frequency (MAF) over 0.4 in the dbSNP Build 135. These designed probes totally covered 761,159 bp of human reference genome (HG19, NCBI build 37). This version called Mini-I was used for our samples from family F-1 and the control sample CG-1, CG-2, CG-3, CP-1 and CP-2. For better coverage of the HBA and HBB gene, we designed an updated version called Mini-II, which was not only covered the HBA and HBB gene region and 4,525 SNPs, but also the 100 K bp region upstream and downstream of the two genes. Mini-II totally covered 1,508,117 bp of human reference genome (HG19, NCBI built 37), and was used in family F-2, F-3, F-4, F-5 and the other control samples.

### Sample Preparation and Sequencing

Genomic DNA (g-DNA) of parents and fetus extracted from peripheral blood and umbilical cord blood and amniotic fluid was fragmented by the sonicator. After end blunted, all fragments were added an “A” tail for the ligation with adaptors. A 6 bp or 8 bp barcode was added to each sample during 12 cycles of PCR for multiple sequencing.

Maternal plasma was isolated using two-step centrifugation protocol [14]. 100 ul maternal plasma was used for DNA extraction and the DNA was fragmented by nature instead of sonication. After “A” tailing, adaptors were also added to each ends of the DNA fragments. For the maternal plasma DNA from family F01, different from adaptors adding to g-DNA, these adaptors were embedded with an 11 bp sequence barcode. There were a total of 384 different barcodes randomly added to each end of each DNA fragment, which could generate 384×384 combinations of labels for original maternal-fetal DNA. Then another 6 bp index was introduced during a 17 cycles of PCR for multiple sequencing. For the other four maternal plasma DNA samples, the adaptors was the same as the gDNA sample, and the 8 bp barcode was added to each sample through a 17 cycles of PCR.

Libraries were qualified by DNA 1000 kit on the Agilent 2100 Bioanalyzer to measure the insert size and quantified by real-time PCR. 3 ug DNA libraries were hybridized with the probes at 65°C for 22 hours. DNA fragments that binding to the probes was eluted and enriched by PCR. Captured DNA libraries were conducted 90 bp paired-end index sequencing on the Illumina Hiseq™ 2000 sequencer according to the manufacturer’s instructions. All of the sequencing raw data had been submitted to NCBI SRA (http://www.ncbi.nlm.nih.gov/sra) and the Submission ID was SRA 065986.

### Alignment and Duplication Reads Removal

Short reads were firstly mapped to the human reference (HG19, NCBI built 37) using SOAP2 [15]with parameters of ‘-v 5–r 1-s 40-l 40’ to select reads with less than one mismatch alignment.

To remove the effect of duplication reads on CNVs detection, we set up a strict criterion to remove duplicated reads introducing from PCR procedure using previous reported UID strategy [12]. For the plasma DNA library constructed using a mixed adaptor with 384 different barcode, the read were considered as duplication, when 1) with the same type of barcode in both reads, and 2) with same starting and end position. For the other samples, the reads meet the condition 2) would be considered as duplication. Only one of them was reserved for the following analysis. Besides that, we also filter regions with less than 10× depth in control samples, which might be related with poor capture efficiency.

### Estimation of cff-DNA Concentration

When we detected the genotype of parents in target region, we used a Bayesian statistical model based on the sequencing depth of each bases. Only different type of homozygous SNPs in both parents was isolated to calculate the fetal concentration in maternal plasma. The formula was
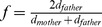
, where 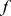 stood for the cff-DNA concentration in maternal plasma, 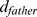 and 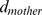
stood for the depth of allele from father and mother respectively. This concentration of cff-DNA would be used for detecting fetal genotype in maternal plasma.

### Fetal CNV Detection

We developed a novel algorithm to identify the fetal CNV combining cff-DNA concentration and sequencing depth in target region (In this case, target region is α-thalassemia related regions described above). The algorithm was mainly consisted of two tests, R-score revealing deviations in the tested sample and L-score representing the probability of fetal CNV comparing to the control samples.

Basic statistics. Before CNV detection, we first defined several basic statistics, including the sequence depth of the 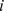-th base of tested sample (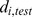) and control sample (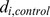). And the relative copy ratio (RCR) was defined as 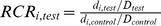, where 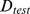
and 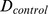 stood for the average sequence depth of test and control sample respectively. Theoretically, RCR represents the CNV genotype of each site in the tested sample. Also, we defined the average sequence depth of each target region (ARCR) as a more stable statistics following 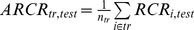 (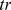 was short for “target region”, 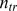 stood for the total base number in this target region). ARCRs of Other sequenced regions except the target region were also calculated.

R-score. R-score is defined as the negative logarithmic probability as 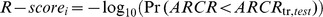. In calculation, we obtained R-score as the quotient between the rank of 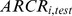 (ranked by algebraic value, from lower value to higher value) and the total number of sequenced regions. For example, if the rank of a tested region is 10, and there are 1000 sequenced regions, the R-score of this region will be 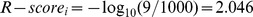. R-score represented the relative relationship between ARCRs, showing their deviation in the tested sample. For example, bigger R-score stands for smaller ARCR, which indicates a potential deletion. In the case of deletion, R-score larger than 1.30 (equals to 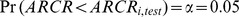) was elected up as one of the cutoff.

L-score. In this test, we recruited a binary assumption strategy to determine the fetal CNV genotype. The L-score was the odd ratio between these two statistics tests of mutually exclusive assumptions, representing the significance of fetal CNV by comparing to the control samples. For better understanding, we took the detection of fetal heterozygous and homozygous deletion as examples. Firstly, we assumed the fetal CNV genotype of this target region was heterozygous deletion (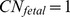, *CN* was short for copy number). The expected copy ratio (ECR) of this region was calculated with the maternal CNV genotype and cff-DNA concentration.



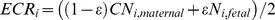
.

RCR value of each site in this region in control samples was normalized to heterozygous deletion. The mean value and standard deviation of all RCRs were calculated as following:
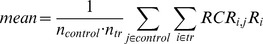


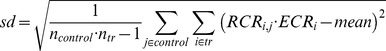



Heterozygous deletion like hood value *l* was then calculated.
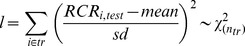



Secondly, we assumed the fetal copy number of this target region was homozygous deletion (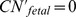). The procedure is totally same as heterozygous assumption.



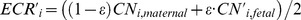
.



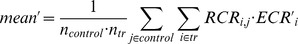
.



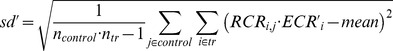
.
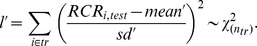



Finally, L-score is defined as the logarithmic quotient between *l* and *l′* as following formula:
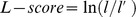



Theoretically, smaller like hood value *l* represent higher similarity between the real fact and the assumption. L-score can represent the deviation of tested sample comparing to controls as CNV. In this sample, L-score was elected up to distinguish fetal heterozygous (L-score<0) and homozygous deletion (L-score>0). Similar detection could be applied to discriminate between heterozygous deletion and normal genotype.

## Supporting Information

Figure S1 **The RCR distribution of target region on HBA.** The color-coded points and lines showed the RCRs distribution of the target regions on HBA gene. RCR value of 0 means a homozygous deletion, 0.5 mean a heterozygous deletion and 1.0 mean normal. The orange broken line represented the mean RCR in maternal plasma. (Gray, 4 plasma control samples respectively; Blue, g-DNA from paternal blood; Red, g-DNA from maternal blood cells; Orange, cell free DNA from maternal plasma; Green, g-DNA from fetal amniotic fluid). (a, F-2; b, F-3; c, F-4; d, F-5).
(TIF)

Methods S1 **Supplementary Methods.**
(DOC)

Table S1 **Performance of target region capture sequencing of control.**
(DOCX)
