# Supplementary figures and images for: Noninvasive Prenatal Detection for Pathogenic CNVs: The Application in α-Thalassemia
Source: PLoS One. 2013 Jun 28;8(6):e67464. doi: 10.1371/journal.pone.0067464 (PMC3696090; doi:10.1371/journal.pone.0067464)

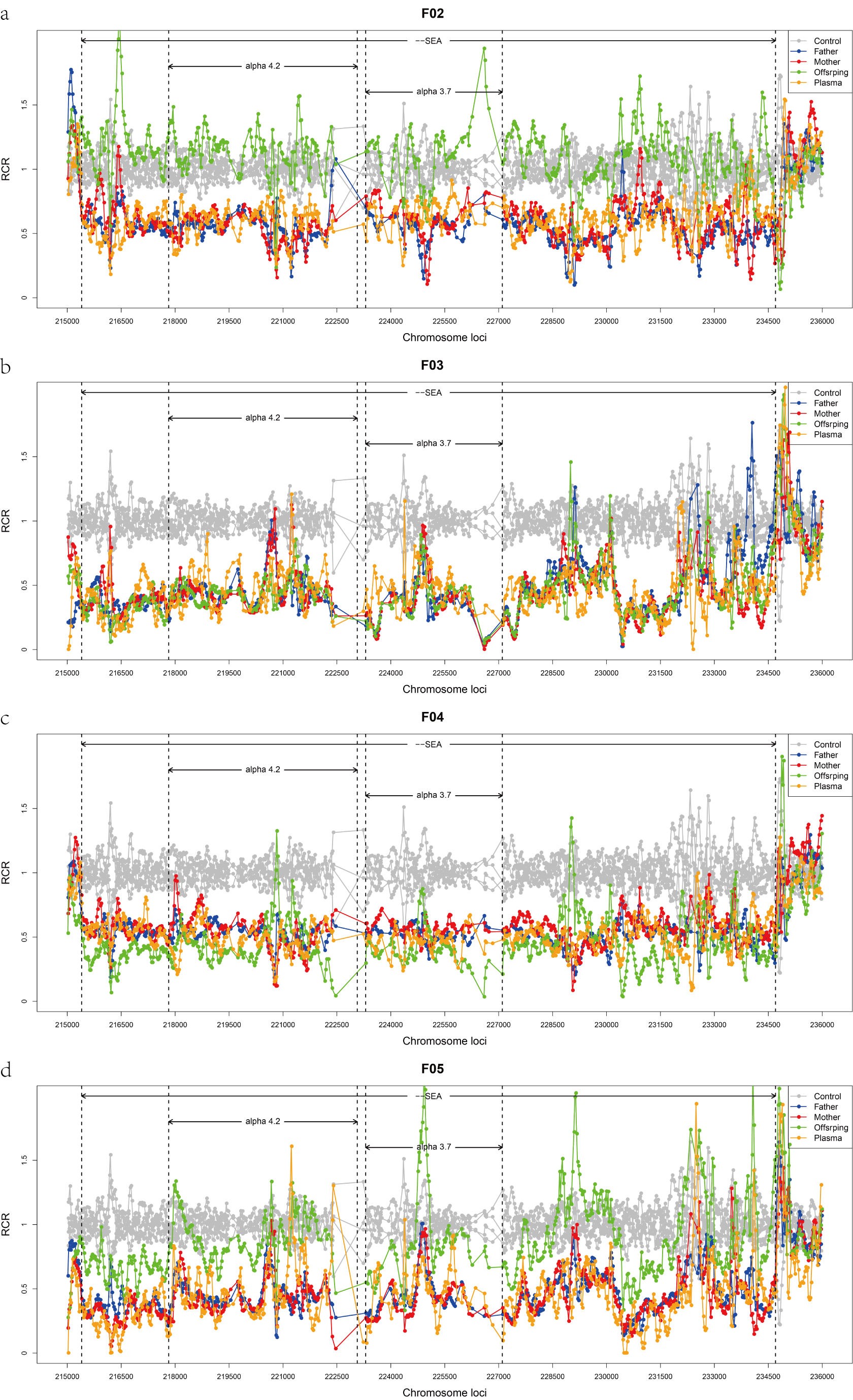

Supplement: Figure S1 — The RCR distribution of target region on HBA. The color-coded points and lines showed the RCRs distribution of the target regions on HBA gene. RCR value of 0 means a homozygous deletion, 0.5 mean a heterozygous deletion and 1.0 mean normal. The orange broken line represented the mean RCR in maternal plasma. (Gray, 4 plasma control samples respectively; Blue, g-DNA from paternal blood; Red, g-DNA from maternal blood cells; Orange, cell free DNA from maternal plasma; Green, g-DNA from fetal amniotic fluid). (a, F-2; b, F-3; c, F-4; d, F-5). (TIF) [file pone.0067464.s001.tif]
