# Supplementary material for: Noninvasive Prenatal Detection for Pathogenic CNVs: The Application in α-Thalassemia
Source: PLoS One. 2013 Jun 28;8(6):e67464. doi: 10.1371/journal.pone.0067464 (PMC3696090; doi:10.1371/journal.pone.0067464)
Supplement: Methods S1 — Supplementary Methods. (DOC) [file pone.0067464.s002.doc]

**Supplementary methods**

Parental CNV calling using target sequencing

For CNV calling using target sequencing with limit target region, we developed a new comprehensive algorithm. In this algorithm, we tested each target region using R-score and L-score to comprehensively determine CNV genotype.

Basic statistics.

Before CNV detection, we first defined several basic statistics, including the sequence depth of the i-th base of tested sample ($d_{i,test}$) and control sample ($d_{i,control}$). Then, we defined the relative copy ratio (RCR) as$RCR_{i,test}=\frac{d_{i,test}/D_{test}}{d_{i,control}/D_{control}}$, where $D_{test}$and $D_{control}$ stood for the average sequence depth of test and control sample respectively. Theoretically, RCR represents the CNV genotype of the tested sample. Also, we defined the average sequence depth of each target region (ARCR) as a more stable statistics following $ARCR_{tr,test}=\frac{1}{n_{tr}}\sum_{i\in tr} RCR_{i,test}$($tr$was short for “target region”, $n_{tr}$stands for the total base number in this target region). ARCRs of Other sequenced regions except the target region were also calculated.

R-score

The R-score is defined as the negative logarithmic probability as$R-score_{i}=-\log_{10}\left(\Pr(ARCR<ARCR_{\text{tr},test})\right)$. In calculation, we obtained R-score as the quotient between the rank of ARCR and the total number of target region. R-score represents the relative relationship between ARCRs, showing their deviation in the tested sample. For example, bigger R-score stands for smaller ARCR. In the case of deletion, R-score larger than 1.30 ($\Pr(ARCR<ARCR_{i,test})=\alpha=0.05$) was elected up as one of the cutoff.

L-score

We defined L-score as the odd ratio between two statistics tests of mutually exclusive assumptions to reveal the significance of tested sample as CNV by comparing to the control samples. In the first test, we assumed the copy number of this target region was normal (diploid, copy number = 2, and the expected RCR = 1).

$$mean=\frac{1}{n_{control}\cdot n_{tr}}\sum_{j\in control} \sum_{i\in tr} RCR_{i,j}$$

$$sd=\sqrt{\frac{1}{n_{control}\cdot n_{tr}-1}\sum_{i\in tr} \left(RCR_{i,test}-mean\right)^{2}}$$

$$l_{1}=\sum_{i\in tr} \left(\frac{RCR_{i,test}-mean}{sd}\right)^{2}\sim\chi_{(n_{tr})}^{2}$$

In the second test, we assumed the copy number of this target region was abnormal, for instance, heterozygous deletion (copy number = 1, and the expected RCR = 0.5).

$$mean'=\frac{1}{n_{control}\cdot n_{tr}}\sum_{j\in control} \sum_{i\in tr} 0.5\times RCR_{i,j}$$

$$sd=\sqrt{\frac{1}{n_{control}\cdot n_{tr}-1}\sum_{i\in tr} \left(0.5\times RCR_{i,test}-mean\right)^{2}}$$

$$l_{2}=\sum_{i\in tr} \left(\frac{RCR_{i,test}-mean'}{sd'}\right)^{2}\sim\chi_{(n_{tr})}^{2}$$

Finally, L-score is defined as the logarithmic quotation between l2 and l1 as following formula.

$$L-score=\ln\left(l_{1}/l_{2}\right)$$

Theoretically, L-score represent the deviation of tested sample from controls as CNV, and L-score>0 was elected up as one of the cutoff for CNV genotype determination.
