# Supplementary material for: Noninvasive Prenatal Detection for Pathogenic CNVs: The Application in α-Thalassemia
Source: PLoS One. 2013 Jun 28;8(6):e67464. doi: 10.1371/journal.pone.0067464 (PMC3696090; doi:10.1371/journal.pone.0067464)
Supplement: Table S1 — Performance of target region capture sequencing of control. (DOCX) [file pone.0067464.s003.docx]

**Table S1. Performance of target region capture sequencing of control samples**

| **Sample** | **Reads (M)** | **Data (MB)** | **Alignment (%)** | **Coverage (%)** | **Depth** |
| --- | --- | --- | --- | --- | --- |
| CG-1 | 2.51 | 0.23 | 85.50 | 92.38 | 106.62 |
| CG-2 | 2.69 | 0.24 | 89.32 | 93.26 | 125.54 |
| CG-3 | 2.35 | 0.21 | 89.17 | 95.33 | 118.13 |
| CG-4 | 21.61 | 1.94 | 92.54 | 96.98 | 88.08 |
| CG-5 | 20.18 | 1.82 | 92.51 | 98.28 | 81.62 |
| CG-6 | 40.22 | 3.62 | 90.84 | 97.30 | 199.62 |
| CG-7 | 27.30 | 2.46 | 90.99 | 98.42 | 145.71 |
| CG-8 | 31.88 | 2.87 | 91.32 | 97.52 | 162.97 |
| CG-9 | 30.27 | 2.72 | 91.22 | 98.54 | 163.97 |
| CG-10 | 31.50 | 2.84 | 92.08 | 97.34 | 186.49 |
| CG-11 | 24.59 | 2.21 | 92.26 | 98.34 | 139.91 |
| CG-12 | 26.91 | 2.42 | 92.64 | 97.06 | 98.25 |
| CP-1 | 127.50 | 11.47 | 90.89 | 95.70 | 44.36 |
| CP-2 | 49.24 | 4.43 | 90.89 | 94.02 | 39.06 |
| CP-3 | 195.05 | 17.55 | 95.62 | 98.70 | 121.24 |
| CP-4 | 177.60 | 15.98 | 95.39 | 97.58 | 154.55 |
| CP-5 | 161.80 | 14.56 | 95.25 | 98.75 | 110.61 |
| CP-6 | 183.83 | 16.55 | 95.33 | 98.73 | 105.25 |
